# Modeling the Dynamics of Disease States in Depression

**DOI:** 10.1371/journal.pone.0110358

**Published:** 2014-10-17

**Authors:** Selver Demic, Sen Cheng

**Affiliations:** 1 International Graduate School of Neuroscience, Bochum, Germany; 2 Mercator Research Group “Structure of Memory”, Bochum, Germany; 3 Faculty of Psychology, Ruhr University Bochum, Bochum, Germany; National Cheng Kung University Medical College, Taiwan

## Abstract

Major depressive disorder (MDD) is a common and costly disorder associated with considerable morbidity, disability, and risk for suicide. The disorder is clinically and etiologically heterogeneous. Despite intense research efforts, the response rates of antidepressant treatments are relatively low and the etiology and progression of MDD remain poorly understood. Here we use computational modeling to advance our understanding of MDD. First, we propose a systematic and comprehensive definition of disease states, which is based on a type of mathematical model called a finite-state machine. Second, we propose a dynamical systems model for the progression, or dynamics, of MDD. The model is abstract and combines several major factors (mechanisms) that influence the dynamics of MDD. We study under what conditions the model can account for the occurrence and recurrence of depressive episodes and how we can model the effects of antidepressant treatments and cognitive behavioral therapy within the same dynamical systems model through changing a small subset of parameters. Our computational modeling suggests several predictions about MDD. Patients who suffer from depression can be divided into two sub-populations: a high-risk sub-population that has a high risk of developing chronic depression and a low-risk sub-population, in which patients develop depression stochastically with low probability. The success of antidepressant treatment is stochastic, leading to widely different times-to-remission in otherwise identical patients. While the specific details of our model might be subjected to criticism and revisions, our approach shows the potential power of computationally modeling depression and the need for different type of quantitative data for understanding depression.

## Introduction

Major depressive disorder (MDD) affects around 20% of the population at some point during the life time of an individual [1]–[4]. Depression is a common and costly disorder that is usually associated with severe and persistent symptoms leading to important social role impairment, increased medical co-morbidity and mortality [5]–[7]. Depression can strike anyone regardless of age, ethnic background, socioeconomic status or gender [8], [9]. According to the World Health Organization, MDD is currently the leading cause of disease burden in North America and the 4th leading cause worldwide [5], [10]–[12]. The onset of MDD is usually between the ages of 20 and 30 years and peaks between 30 and 40 years [13], [14].

The understanding of the nature and causes of depression has evolved over the centuries, though this understanding is incomplete and has left many aspects of depression as a subject of discussion and research. The heterogeneity of depression implies that multiple neural substrates and mechanisms contribute to its etiology [15]. Proposed causes include psychological, psychosocial, hereditary, evolutionary and biological factors. Family, twin, and adoption studies provide evidence that genetic factors might account for some risk of developing MDD [16]–[18]. According to diathesis-stress theories of depression, genetic liability (diathesis) interacts with negative life experiences (stress) to cause depressive symptoms and disorders [19], [20]. Indeed, there is some evidence for the involvement of specific genes and gene-by-environment interactions in the pathogenesis of MDD, even though they cannot account for all occurrences of MDD [5].

One major theory about the biological etiology of depression suggests that the underlying pathophysiological basis of depression is a depletion of the neurotransmitters serotonin, nor-epinephrine or dopamine in the central nervous system [21]–[23]. Although most antidepressants drugs (ADs) produce a rapid increase in extracellular level of the monoamines, the onset of an appreciable clinical effect usually takes at least 3–4 weeks [24]–[29]. This delayed onset of action, or response, which is usually defined as a 50% reduction in depression rating scale score compared to baseline [30], suggests that dysfunctions of monoaminergic neurotransmitter systems found in MDD represent the downstream effects of other, more primary abnormalities. In addition, success of AD treatment is relatively low. Selective serotonin re-uptake inhibitors (SSRIs) are frequently used as a first medication for MDD, but have response rates of 50% to 60% in daily practice [31]–[35]. In some studies, ADs even fail to show superiority over placebo [36]–[38]. More precisely, the response to inert placebos is approximately 75% of the response to active AD medication [36], [39]. The high rate of inadequate treatment of the disorder remains a serious concern. Research comparing AD medication to cognitive behavioral therapy (CBT) has found that both are equally effective for non-psychotic forms of depression [40]. Indeed, in some theories of depression, cognitive aspects are dominant factors in the etiology and maintenance of the disorder [41]–[43]. These models postulate that depressed patients process depression-congruent information selectively, which seems to form part of a vulnerability factor.

In addition to the heterogeneous etiology of MDD, the disorder shows complex transitions between several disease states. According to the Diagnostic and Statistical Manual of Mental Disorder, 4th edition, text revision (DSM-IV-TR), the standard for the diagnosis of mental disorders, a depressive episode (DE) is characterized as a period lasting at least 14 days, during which the patient is consistently within the symptomatic range of a sufficient number of symptoms [1], [44]. A DE can be interrupted by remission, which is defined as an asymptomatic period of at least 14 days [45]. A remission and recovery are accompanied by the same behavioral symptoms and, at the behavioral level, distinguished only by their duration. A remission that lasts for 6 months or longer is called recovery [45]. This term refers to recovery from the episode, not from MDD per se. The appearance of a new DE after recovery is called a recurrence [45]. A relapse is a return of the symptoms satisfying the full syndrome criteria for an DE during the period of remission, but before recovery [1], [45]. According to a population-based study among depressive patients, about 15% of first lifetime onsets have unremitting course, and 35% recover but have one or more future episodes [46], [47]. These cases may represent chronic and more severe forms of MDD [46], [48]. About 50% of first lifetime onsets recover and have no future episodes [46]. However, the disease states in depression are not defined consistently by different investigators, thus making it difficult to interpret the results and precluding comparisons between different studies.

All hypotheses that try to explain the dynamics of depression have certain limitations, so our understanding what causes depression is still incomplete. Existing hypotheses are not exclusive, but rather complementary. The question is how to integrate the different hypotheses. Mathematical models are well-suited for this problem. Here, we aim to systematically define the states in the course of MDD and to study the dynamics of MDD. We developed a single abstract model that is consistent with many existing theories about depression. Although our model is not mechanistic, it helps us to understand and analyze the etiology and dynamics of MDD. Finally, we modeled the influence of three types of therapies (antidepressant treatment, cognitive behavioral therapy, and life style changes) on the occurrence and duration of depressive episode.

## Methods

### Dynamical systems model of major depressive disorder

To model the dynamics of depression, we first need a way to describe the state of a person, i.e., whether a person is suffering from MDD or not. We adopted the simplest approach possible, which is to describe the state of a person by a single variable. We call this variable *M*, loosely for mood. *M<0* indicates that the person suffers from symptoms associated with MDD; the person is in the symptomatic state. In our simple model with only one variable, we do not model which precise symptoms patients suffer from. A negative value of the state variable indicates that the person satisfies a sufficient number of symptoms (Table 1) to meet the syndromal criterion for a depressive episode according to DSM-IV-TR [1]. If this state persists for fourteen days or more, the person is considered to suffer from MDD [1], [45]. *M>0* indicates that the person does not meet the syndromal criterion for a depressive episode; the person is in the asymptomatic state.

**Table 1:** DSM-IV-TR Criteria for Major Depressive Disorder [1].

Five or more of the following symptoms should be present daily for most of the day for at least 2 weeks
At least one symptom is either depressed mood or anhedonia
Changes in appetite or weight
Insomnia or hypersomnia
Psychomotor agitation or retardation
Fatigue or loss of energy
Feelings of guilty or worthlessness
Difficulty with thinking, concentrating, or making decisions
Suicidal ideation or suicidal attempts

The variable *M* changes across time to account for changes in the symptoms and progression of MDD. We model the time evolution of *M* in discrete time steps according to this simple equation



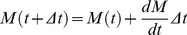
(1).

**Figure 1:**
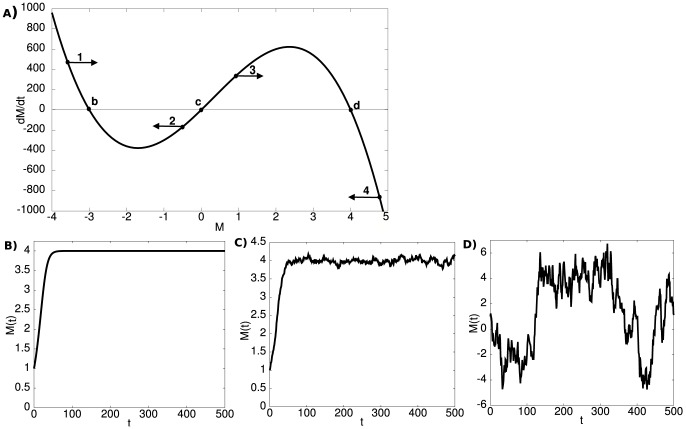
Dynamical systems model for the dynamics of mood. **A**) A schematic showing the mood change as a function of the state variable *M* without external inputs and noise (*I = ε = 0*). The arrows at 1, 2, 3, 4 indicate the direction of change in those states. The points labeled with *b, c,* and *d* are fix points. At these points, the value of the change is zero (dM/dt  = 0). Therefore, when there is no noise, the state will not change once it has reached a fix point. The fix points *b* and *d* are stable, meaning that the system will return to these states if slightly perturbed. The fix point *c* is unstable and has different properties, the system will move further away from point *c* even if the system is only slightly perturbed. In that case, the system will evolve until it reaches one of the stable fixed points. If 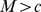, the system will move towards the fix point *d*. The system will evolve towards the other fix point *b*, if 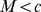. Therefore, the fix point *c* separates the basins of attraction of the two stable fix points. Samples of the evolution of *M* over time **B**) without noise, **C**) with a moderate level of noise and **D**) with high level of noise. Note, that with high level of noise the system exhibits stochastic transition between positive and negative values.



In each time step 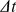, the mood changes by the amount 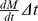. The crucial issue is how to model the dynamics of the mood given by 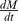. The dynamics fully determines the behavior of the system and should account for the major empirical observations in MDD as outlined in the Introduction. We were looking for a simple model that can capture many of the important clinical observations related to MDD. The simplest model is a linear one with a single stable point. Preliminary work showed that linear dynamics does not account for many important observations. It was too easy to switch from positive to negative mood and vice versa, which is in contradiction with the phenomenology of MDD. Thus it was evident that we needed a model that has two stable states, one corresponding to a depressive state and the other to a non-depressive state. We therefore chose to model the dynamics with a polynomial of third degree (Fig. 1A).

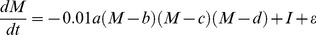
(2),where *a>0; b, c, d* are parameters to be studied, *I* is an external input, and ε is a Gaussian noise term with zero mean and a standard deviation of one to set the scale. In our model, the system is driven both by deterministic intrinsic dynamics (cf. Fig. 1B) and a stochastic noise process (cf. Fig. 1C, D). The intrinsic dynamics is an abstract model of the changes in the mood of a person driven by deterministic physiological processes, processes which we do not attempt to model mechanistically here. The dynamical system in Eq. (2) has two stable fix points, separated by an unstable fix point. The parameters *b, c*, and *d* are ordered such that 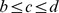 (Fig. 1A). The parameters of the model specify the unique dynamics of a system, which represents a person. Depending on how the parameters affect the dynamics of MDD, we assign them to possible physiological correlates (Table 2). Within a subpopulation in our model, all individuals share identical parameters. By contrast, the noise process captures stochastic physiological processes as well as external environmental factors. Fluctuations in the mood can be caused, for instance, by random hormonal changes or by changes due to the circadian rhythm. Also, external changes might cause fluctuation in the mood of a person during the day, i.e., stressful situations at work or rapid weather changes. The name “noise” does not imply that the noise process is irrelevant or unimportant. On the contrary, the noise term is crucial in our model since it introduces unpredictable changes to the mood. This stochasticity is what makes the time-course of the mood of one modeled person (cf. Fig. 1D) different from that of another person.

**Table 2:** Potential physiological correlates of the model parameters.

Symbol	Parameter	Potential physiological correlates
*a*	Decay rate	Hippocampal volume and rate of adult neurogenesis
*b*	Negative stable fix point	Level of monoamines (i.e. serotonin)
*c*	Instable fix point	Pessimistic attitude c>0, optimistic attitude c<0
*d*	Positive stable fix point	Amygdala activity (higher activity is represented by a smaller d)
*I*	External input	Environmental influence
*ε*	Noise	Unpredictable internal or external changes that cause fluctuation in the mood

### Relating occurrence and recurrence rates to the distribution of the number of depressive episodes

We use empirical occurrence and recurrence rates to compute the distribution of the number of depressive episodes during an individual's lifetime (NDE) since the latter is rarely reported by epidemiological studies but quite informative. The occurrence rate (OR) is the fraction of the population that suffers from at least one DE during their life time. The OR thus equals the probability of having one or more depressive episodes, i.e.,
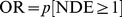
(3)


The rate of first recurrence 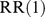 is the fraction of patients who suffer from depression a second time out of those patients who suffered from one previous depressive episode. The rates of second recurrence 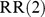, third recurrence 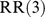, etc. are defined similarly. In general, 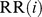 can be calculated using the following equation
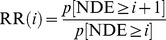
(4)


The probability of having no depressive episode is



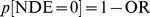
(5).

The probability of having exactly one depressive episode is



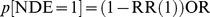
(6).

The probability of having two or more depressive episode is computed according to this equation
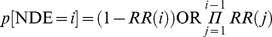
(7)for 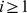.

To study the OR and RR, we initialized the system in a positive state 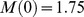 and simulated the dynamics of MDD for a period of 70 years using a time-step 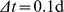. For both the single population and the two sub-populations model, the analyses are based on simulations of 10000 individuals. In the two sub-populations model, *93%* of individuals belong to the low-risk sub-population while the remaining *7%* belong to the high-risk sub-population.

### Studying treatment effects

To study the time-to-remission and time-to-response in our model, we initialized the system in a negative state 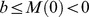. The initial value was drawn from a uniform distribution. We simulated the dynamics of MDD for a period of 20 years using a time-step 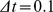
d. To study the effects of different treatments, we used only simulations of the two-subpopulations model in which a DE occurred. Hence, *63%* of the simulations belong to the low-risk sub-population while the remaining 37% belong to the high-risk sub-population. We initialized the finite-state machine in the rebound depressive episode state and considered the time when *M* increases above 50% as time-to-response, and the time at which the first remission or recovery occurred as the time-to-remission. Fourteen days were added to the time-to-remission to account for the fact that symptoms have to be present for at least fourteen days to qualify as an DE. In the control group, parameters were identical to those used for the simulation of the occurrence rate. In the experimental group, we changed certain parameters in order to simulate the effect of various treatments such as AD treatment (change in parameter *a*, and *d* or *b*), CBT (change in parameter *c*) and life style changes (change in parameter *I*).

All simulations and analyses were performed in Matlab R2012a (MathWorks; Natick, Massachusetts, USA) using custom-written software. The code is freely available online at http://cns.mrg1.rub.de/index.php/software

## Results

### Finite state machine: systematic definition of disease states

**Figure 2:**
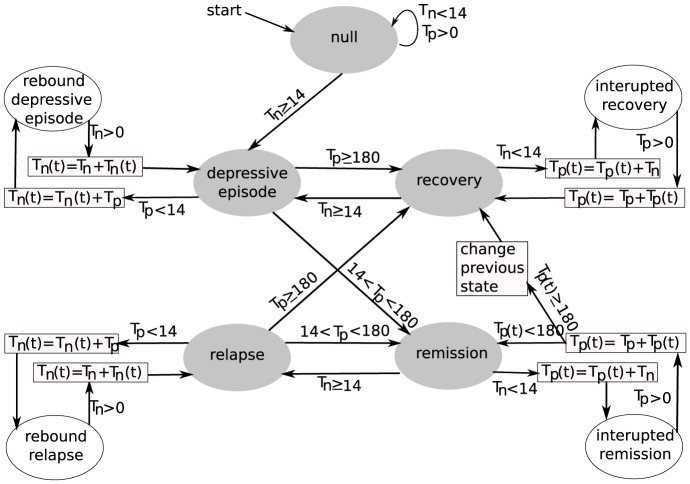
Finite state machine modeling the transitions between the disease states in depression. State diagram for the finite state machine. Ellipses represent the disease states in depression. Grey filled ellipses are clinically relevant disease states; unfilled ellipses are auxiliary disease states that are needed to discount short interruptions of clinically relevant disease states. The arrows indicate transitions between disease states. Transitions only occur when the state variable *M* changes sign, i.e., either from positive to negative, or vice versa. Each arrow is labeled by the criteria that trigger the transition. 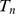 represents the length (in days) of the period during *M<0* before transition to a positive value occurred. In other words, 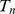 is the duration that a person meets the syndromal criterion for a depressive episode according to DSM-IV-TR [1]. Accordingly, 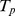 represents the length (in days) of the period during *M>0*, i.e., the duration in which a person does not meet the syndromal criterion for a depressive episode. The rectangles indicate a change to previously identified states. Short interruptions of disease states are added to the duration of disease states.

**Figure 3:**
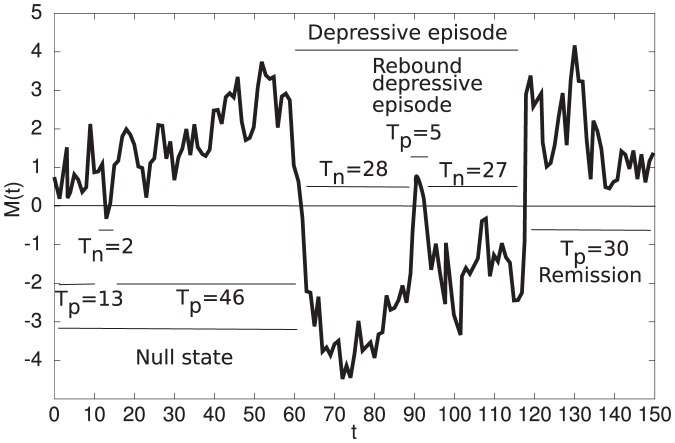
Example of the time course of the state variable *M* and the disease states identified by the finite state machine. In this example, a symptomatic period lasting 28d is interrupted by an asymptomatic period of 5d and followed by another symptomatic period of 27d. Therefire, our model identifies the three periods together as a single depressive episode of length 60d. 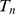 and 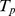 represent the length (in days) of the period when *M*<0 and *M*>0, respectively.

We developed a finite state machine (Fig. 2) to systematically define the disease states of MDD: *depressive episode, remission, recovery*, and *relapse*, which were described above. This mathematical model analyzes the transitions of *M* from the asymptomatic to the symptomatic state, and vice versa, and assigns a disease state to each time interval. The disease state changes depending on the length of periods for which *M* remains positive (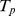) or negative (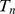) (see Fig. 3 for an example). In addition to the disease states of clinical relevance, we had to introduce auxiliary disease states to account for short interruptions of a disease state that are clinically irrelevant. For instance, if a one-month-long depressive episode is interrupted by a 2-day-long period in the asymptomatic state, there is little reason to assume that the short interruption has any relevance. Clinicians frequently make such intuitive judgments without making them explicit [45], but such discounting has to be build in explicitly in a mathematical model.

In the following, we describe the auxiliary disease states in more detail. The *null state* is the initial state, before any data is available to make a more specific determination of the disease state. Short periods in the symptomatic state, 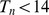, and any duration in the asymptomatic state, 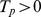, will not change this state (Fig. 2&3). The only possible transition out of the null state is to a DE, if the syndromal criterion is met for at least 14 consecutive days, i.e., 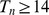. A *rebound depressive episode* is an interruption of a DE that is shorter than two weeks (Fig. 2&3). The duration in the positive state is added to the duration of the DE (boxes connected to rebound depressive episode in Fig. 2). Similarly, *rebound relapse, interrupted remission*, and *interrupted recovery* are interruptions of the relapse, remission and recovery states of MDD, respectively. The auxiliary disease states are necessary to discount short interruptions of the disease states in our model and have little clinical relevance. We therefore focus our attention on the clinically relevant disease states in the following.

One point requires special attention. Recovery occurs after an asymptomatic period of 6 months or more, even if that period is interrupted by short periods (<14 days) in the negative state. If the first period in the positive state lasts for longer than 14 days and less than 6 month, then the finite-state machine will initially label this period as remission. Short interruptions in the negative state and the following periods in the positive state are added to duration of remission. If the total duration of the “remission” period exceeds 6 months, then the period becomes recovery. To correct the initial classification, we included an action “change previous state” (Fig. 2). This finite-state machine unambiguously defines the disease states and can be used to track their evolution over the lifespan of patients as well as in our theoretical simulations.

### Dynamical systems model for the dynamics of major depressive disorder

We developed a simple dynamical systems model (see Methods) to simulate and study the progression of disease states over 70 years. In a first attempt, we speculated that perhaps all people share the same dynamics parameters, and thus similar physiological parameters, and that depressive episodes occur stochastically.

**Figure 4:**
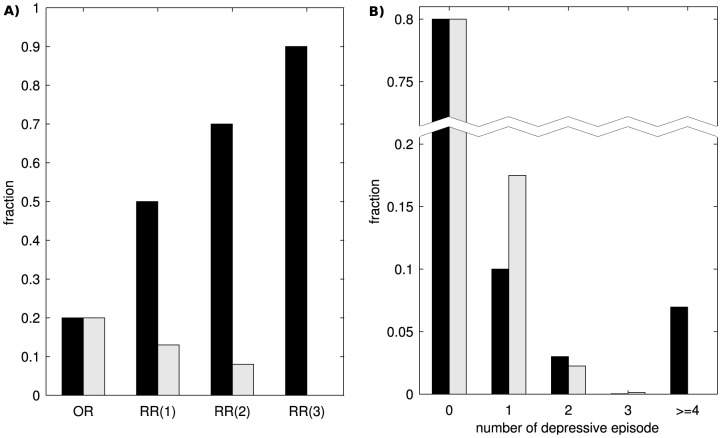
Single population model can account for empirical occurrence rate but not for recurrence rates. **A**) The occurrence rate (OR) from our simulation (grey bars) was fit to the result from epidemiological studies (black bars). The parameters of the model are: *a = 4.65; b = −3; c = 0.175; d = 5; I = 0.02*. However, in our simulation, the recurrence rates, RR(i), decrease with the number of prior depressive episodes, which is contrary to epidemiological data. **B**) The distribution of the number of depressive episodes (DE). The probability of zero DE is 0.8. The bars were cut off to show more clearly the smaller probabilities for the higher numbers of DE. The epidemiological distribution is clearly bimodal (black bars), whereas the simulated distribution is unimodal (grey bars).

If our model captures some aspect of the dynamics of MDD, it should be able to account for the epidemiological data on occurrence and recurrence rates of MDD (see Methods). According to DSM-IV-TR, *OR = 20%*, *RR(1)* = *50%*, *RR(2) = 70%*, and *RR(3) = 90%* [1]. In our first modeling attempt, we chose a single set of parameters representing a homogeneous population to match the epidemiological occurrence rate. The parameters of the single population model were: *a = 4.65; b = −3; c = 0.175; d = 5; I = 0.02*. However, this model does not match any of the epidemiological recurrence rates (Fig. 4A). The mismatch is not simply a numerical issue, the model yields qualitatively different data. Rather than having rates that increase with the number of DE as in epidemiological studies, in our single-population model, the rates decrease.

**Figure 5:**
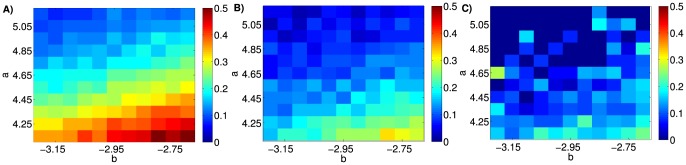
Influence of parameters a and b on the occurrence and recurrence rate in the single population model. **A**) Occurrence rate, **B**) first recurrence rate, and **C**) second recurrence rate, each represented by color scales, for a range of the parameters *a* and *b*. The remaining parameters are: *c = 0.175; d = 5; I = 0.02*. Note, that for all combinations of the parameters *a* and *b*, the rate of first recurrence is lower than the occurrence rate, and the rate of second recurrence is lower than the rate of first recurrence.

This is not surprising. Since a one-dimensional model has no memory other than the current state, the probability of the second DE (the first recurrence) occurring within a certain time period is the same as the probability of the first DE. However, the first DE can occur anytime within the full 70 years of simulated time whereas the second DE can only occur after the first DE had already occurred. Since the number of DE are proportional to the length of the observation period, the first recurrence rate is lower than the occurrence rate. The same logic applies to the second and third recurrence rates, which are successively lower (Fig. 4A). Our argument implies that this property is not specific to the particular parameters that we chose. Indeed, additional simulations show for a range of the parameters *a* and *b* that, in the single population model, the rate of first recurrence is lower than the occurrence rate, and the rate of second recurrence is lower than the rate of first recurrence (Fig. 5). To further investigate how the single population model deviates from the true dynamics of MDD, we calculated the distribution of NDE (Fig. 4B). In the simulated data, the likelihood monotonically decreases such that four or more DE are absent from our simulated data. In contrast, the epidemiological data show that four or more DE occur with a substantial probability of around *7%* and is even higher than the probability of two or three DE. Since the epidemiological data follows a bimodal distribution, we hypothesized that two subpopulations might be required to account for empirical occurrence and recurrence rates of MDD.

**Figure 6:**
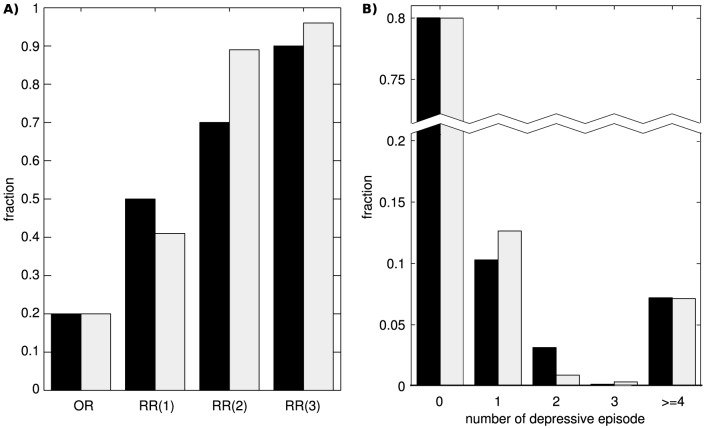
Two sub-population model can account for empirical occurrence and recurrence rate. **A**) The parameters of the two sub-population model are: *a = 5; b = −2.85; c = 0.175; d = 5; I = 0.02* for the low-risk sub-population and *a = 4.4; b = −3.75; c = 0.175; d = 4.25; I = 0* for the high-risk sub-population. Our simulation data (grey bars) closely matches the empirical (black bars) occurrence and recurrence rates and **B**) the distribution of the number of depressive episodes.

We therefore simulated data for two sub-populations. In this model, ninety-three percent of the population shares low-risk parameters and develops depression with low probability. The parameters of this sub-population were chosen (*a = 5; b = −2.85; c = 0.175; d = 5; I = 0.02*) such that *OR = 13%*. The remaining seven percent of the population belongs to the high-risk sub-population and develops depression with very high probability (*∼100%*). The parameters for this sub-population were: *a = 4.4; b = −3.75; c = 0.175; d = 4.25; I = 0*. At this point, we would like to stress that the two sub-populations together represent the entire population, which implies that no one is absolutely immune to depression. By design, the two sub-population model yields a bimodal distribution of NDE (Fig. 6B). With this two sub-population model, we were able to match the empirical occurrence and recurrence rates of MDD (Fig. 6A).

### Modeling the effect of antidepressant treatment

**Figure 7:**
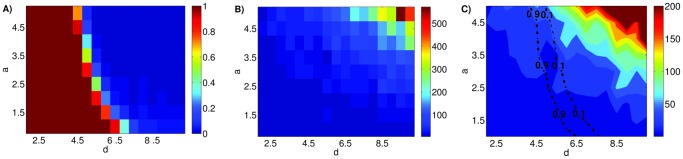
Modification of parameters a and d cannot account for the effect of antidepressant treatment. Shown in the color scales are the occurrence rate (**A**), the median time-to-remission (**B**) and the contours of the median time-to-remission (**C**) in simulated data. Consistent with the assumption that monoamine levels correlate with parameter *d* and the rate of adult neurogenesis with parameter *a*, the occurrence rate decreases with increasing parameters *a* and *d* (A). However, modeling the effect of antidepressant treatment as increases in parameters *a* and *d* would make the paradoxical prediction that antidepressant treatment increases the time-to-remission (B). **C**) To show this conflict more explicitly we plot both the occurrence rate and the time-to-remission in the same panel. The dashed lines represents contours in the occurrence rate at the indicated values, while the color scale represents median time-to-remission. It is highly unlikely to find parameter combinations of *a* and *d* which reduces the time-to-remission while keeping the occurrence rate constant or lowering it.

**Figure 8:**
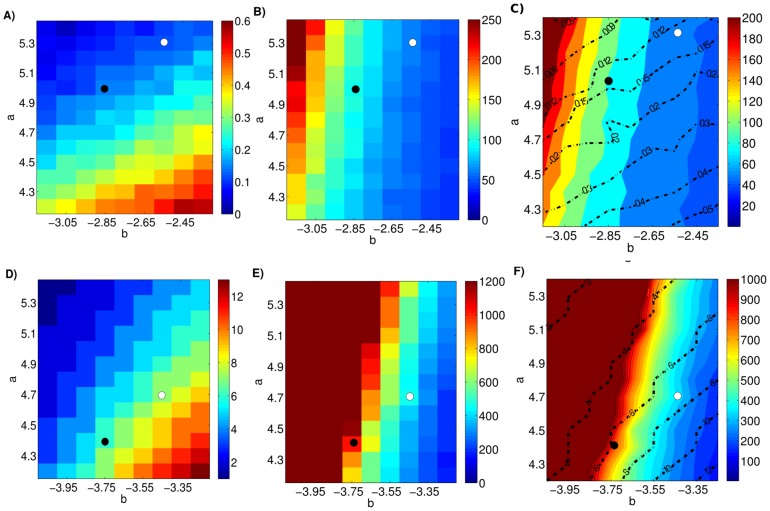
Increases in parameters a and b are consistent with the effect of antidepressant treatment. The first row of panels shows the results of simulations for the low-risk sub-population where the color scales in **A**) and **B**) represent the occurrence rate and median time-to-remission, respectively. Panel **C**) displays the same data using contour lines (occurrence rate) and color scale (media time-to-remission). The second row of panels shows the results for the high-risk sub-population where the color scale represents **D**) the median number of depressive episodes and **E**) median time-to-remission. Panel **F**) displays the same data using contour lines (median number of depressive episodes) and color scheme (median time-to-remission). The black and white points mark pre- and post-treatment parameters, respectively. For certain parameter combinations an increase in the parameters *a* and *b* reduces the median time-to-remission while keeping the occurrence rate (the median number of depressive episodes for the high risk sub-population) constant or lowering it.

**Figure 9:**
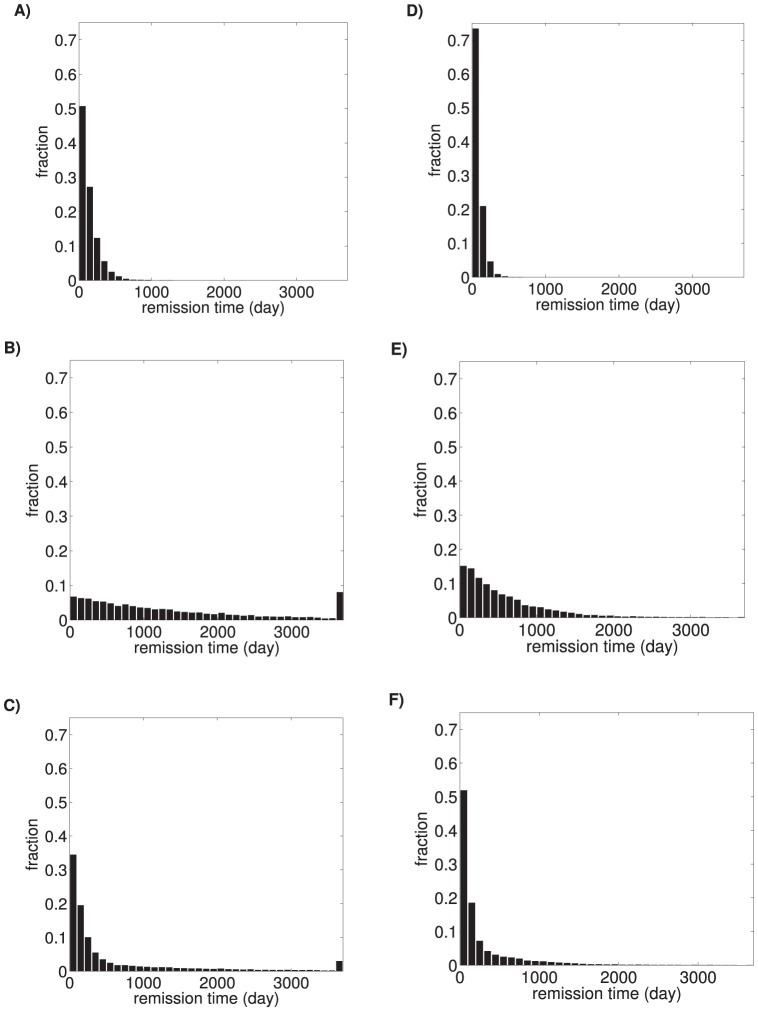
Distribution of the duration of depressive episodes. A), B), and C) show data for control group with pre-treatment parameters. D), E), and F) show data for treatment group with post-treatment parameters. The first row (A, D) of panels shows the duration of depressive episodes for the low-risk subpopulation, the second row (B, E) for the high-risk subpopulation, and the third row (C, F) for the joint distribution. Note that the distributions have long tails, indicating that some patients take much longer to improve than others, even though they all share the same parameters.

The most commonly used antidepressants are those that regulate the metabolism of monoamines in the brain, in particular serotonin. Our initial hypothesis was that the parameter *d* correlates with monoamine levels. Furthermore, it was shown that treatment with AD increases the rate of adult neurogenesis in the dentate gyrus [49] and it has been suggested that adult neurogenesis is important for memory [50]. Since the parameter *a* determines how quickly the current state is forgotten, we hypothesized that AD treatment increases parameter *a*. Since the intended effect of AD treatment is to reduce the time that patients suffer from the symptoms of MDD, we decided to use the time-to-remission as the target parameter for AD treatment. Our simulation results contradict our initial hypothesis, increasing the parameters *a* and *d* increases, rather than decreases, the time-to-remission (Fig. 7B&C). One potential resolution could be to assume that we correctly guessed the physiological correlates of the parameters a and d, but the relationship is inverse to our expectation. However, this interpretation is inconsistent with the OR in our simulations. Decreasing parameters a and/or d, increases the OR. Thus if we modeled the effect of AD treatment as a decrease in parameters a and/or d, it would imply paradoxically that AD treatment of healthy patients increases the OR of MDD (Fig. 7A, C). An extensive parameter search did not yield any parameter changes in *a* and *d* that have the desired change time-to-remission and OR simultaneously. We therefore turned to model the increase in the level of monoamines as an increase in parameter *b* (Fig. 8). In this scenario, the time-to-remission is reduced by an increase in b, but elevated by an increase in a (Fig. 8B, E). While the latter outcome is an undesirable property, there are combinations of simultaneous increases in parameters a and b that yield a lower time-to-remission. This is possible because the contour lines are not parallel to the axes or, in other words, the parameters are inter-dependent. Similarly, the OR is reduced by an increase in *a*, as desired, but elevated by an increase in *b* (Fig. 8A, C). Again, there are combinations of simultaneous increases in parameters *a* and *b* that yield a lower occurrence rate. Importantly for the change of parameters indicated by the black and white points, representing pre- and post-treatment parameters, the change in both the time-to-remission and occurrence rate are in the desired directions. We therefore suggest that parameter a correlates with the rate of adult neurogenesis and parameter b with monoamine levels (Table 2). It is worthwhile to note that AD treatment in our model does not work like a deterministic switch. Even though AD treatment in our model alters the physiological parameters immediately, remission remains a stochastic process driven by the intrinsic dynamics and the noise term. The results of our model demonstrate that the time required to see a significant effect of antidepressants is about three weeks, which is highly similar to the epidemiological data (see Table 3). Figure 9 shows the distribution of the duration of DEs. Both the treated (Fig, 9D, E, F) and control groups (Fig. 9A, B, C) exhibit distributions with large variances and long tails. This result is somewhat surprising given that within each subpopulation all individuals share the same parameters and it underlines the difficulty in understanding the physiological mechanisms of AD treatment. These highly skewed distributions might explain why the median duration of depressive episodes reported in the literature varies widely from three to twelve months, even if most studies suggest that the median duration of depressive episode is about three months [51]–[56]. Overall, we find that our model reproduces rather well other variables which are often used in a clinical and epidemiological studies to examine the efficacy of AD treatment (Table 3).

**Table 3:** Comparison of quantitative measures of disease progression between model and clinical observation.

Variable	Observations	Model
*Occurrence rate, OR*	20% [1], [82], [85]	20%
*1st recurrence, RR(1)*	50% [1], [85]	40%
*2nd recurrence, RR(2)*	70% [1]	90%
*3rd recurrence, RR(3)*	90% [1]	96%
*Mean time-to-response*	2 to 3 we [26], 3 to 4 we [24], 20 to 31 we [87]	20d
*DE duration in patients treated with AD (from onset of DE to remission/recovery)*		
Mean	8.4 mo [51]	267d
Median	3 mo [51], 16 we [82], 22 we (1st DE) [55], 19 we (4th DE) [55]	96d
p(TDE< = 90 d)	50% [51], 53% [60]	48%
p(TDE< = 180 d)	63% [51]	68%
p(TDE< = 360 d)	76% [51]	80%
p(TDE< = 630 d)	80% [51]	87%
p(TDE>720 d)	12% [84],15% [46], 20% [51], 20% [54], 22% [83]	11%
*DE duration in patients treated with AD (from onset of AD treatment to remission/recovery)*		
Mean	5.6 mo [81]	252 d
Median	3 mo [81]	85 d
p(TDE< = 30 d)	26% [81]	24.5%
p(TDE< = 90 d)	63% [81]	52%
p(TDE< = 180 d)	77% [81]	69%
p(TDE< = 360 d)	85% [81]	80%
p(TDE< = 720 d)	88% [81]	87%
*Median age of onset of 1st DE*	early-to-mid twenties [84]	25 y
*DE duration in patients treated with CBT*		
p(TDE< = 3 m)	50% [60]	49%
p(TDE< = 16 week)	52% [86]	57%

**AD**: antidepressant; **DE**: depressive episode; **TDE**: duration of depressive episode

### Modeling the effects of cognitive behavioral therapy and life style changes

**Figure 10:**
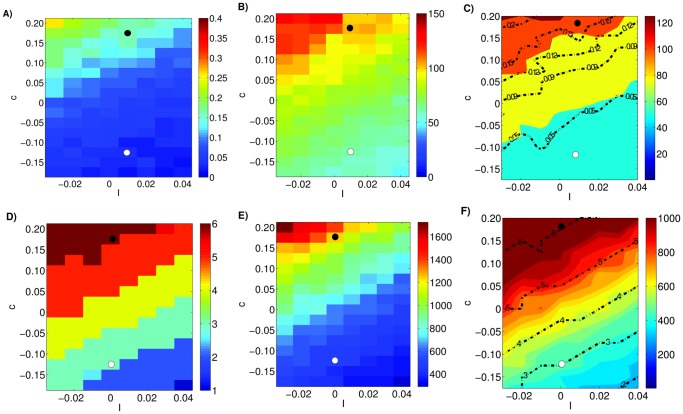
Modeling the effect of cognitive behavioral therapy and life style changes on MDD. Plotting convention as in Figure 8. An increase in the parameter *I* and/or decrease in *c* reduces the occurrence rate (A) (the median number of depressive episodes for the high-risk sub-population, D) and the median time-to-remission (B and E). These results suggests that smaller values of parameter *c* correlates with more positive attitude and larger values of *I* correlate with more positive environmental influences.

CBT instructs patients with MDD to develop a more optimistic approach to life and to detect and transform negative thoughts into positive thinking [40], [57], [58]. The effect of CBT is an improved ability to deal with difficult circumstances and shorter durations of DEs [59], [60]. A similar effect occurs in our simulations when we decrease parameter *c*: both the OR and the time-to-remission decrease (Fig. 10, the black and white points represent pre- and post-treatment value of parameter *c*, respectively). Moreover, the results of our model show that about half of the patients treated with CBT will be in remission after three months of treatment and that the number of patients in remission increases with elapsed time, in line with the epidemiological data (see Table 3). Hence, we hypothesize that smaller c correspond to optimistic attitude and larger c to pessimistic attitude (Table 2).

Life style changes such as, for instance, exercise, social support, and stress reduction lead to a lower probability of having another DE and to shorter duration of DEs, if they do occur [61]–[63]. Indeed, a recent study compared exercise, antidepressant medication and combined medication and exercise in adults and found that all treatments were effective [61], [62]. Since external factors enter our model through the parameter *I*, we suggest that the parameter *I* correlates with environmental influence, where larger *I* corresponds to positive environmental influence and smaller *I* to negative influence (Table 2). Our simulations confirm that increasing *I* indeed decreases the time-to-remission and the OR (Fig. 10). In addition, our results suggest that the combination of the two interventions, CBT and life style changes, will yield better results in the treatment of depression and the prevention of relapses and recurrence than their individual application.

## Discussion

In this article, we have developed a finite-state machine to systematically define the states in the course of MDD together with operational criteria for the terms remission, recovery, relapse, and recurrence. We used a simple dynamical systems model to simulate the day-to-day fluctuations in the mood that might correlate with depression. While this model is not a physiological model, it incorporates several parameters that can be associated with physiological mechanisms. The advantage of this model is that it can incorporate several biological and psychological factors that are thought to affect MDD, and describe their potential interactions. Combining the finite-state machine and dynamical systems model, we studied the dynamics of disease states in depression and found that two sub-populations, one high-risk and one low-risk, are required in our model to account for the empirical data. The two sub-populations model is able to reproduce many, though not all, observations quite well.

One parameter, *d*, we have not associated with a physiological or cognitive roles, yet. The influence that parameter *d* has on the occurrence rate and time-to-remission suggests that *d* might correlate partly with amygdala activity. Indeed, other authors before us have tied the amygdala to depression [64], [65]. Hyperactivity in the amygdala is a common finding during baseline conditions in MDD [66] and has been interpreted as a valence-specific effect that causes a negative memory bias [67], [68].

### Dimensionality of the model and history-dependence

DSM-IV-TR, used worldwide as a diagnostic tool, does not define absolute boundaries between mental disorder and no mental disorder. However, the use of a categorical classification is fundamental in everyday clinical practice and research, as well as for health services and insurance purposes. The categories are prototypes, which define certain criteria related to symptoms, i.e., we say that a patient with a close approximation of the ill-prototype is ill. However, it has been argued that MDD should not be treated as a categorical condition and instead be viewed along a continuum [69]. Instead of categorizing subjects as ill or healthy, they should be scored on a graded scale according to how may symptoms the subject expressed and/or how severe the symptoms are. Some authors go even further to suggest that a one-dimensional approach is not sufficient and that multiple dimensions have to be used to capture the multiple facets of depression. In this study, we reject the view that depression is a categorical condition, and model the dynamics of MDD with a continuous state variable (*M*). However, since virtually all existing observations on MDD have been based on categorical classification and clinical practice depends on it, we developed the finite-state model to translate between the dynamics of a continuous one-dimensional system and the categorical classification of disease states. Since we are at an early stage of the modeling process, it appeared prudent to start with a single state variable to model the dynamics of MDD, especially given the paucity of data that could constrain higher-order systems. In addition, the general approach in modeling is to start with a parsimonious model and to include more complexity only if and when additional mechanisms are required. So far, the simple model we studied has been able to account for a surprisingly wide range of observations.

A consequence of the choice of a one-dimensional model is that, at a given point in time, the system's behavior is fully determined by the one state variable. As a result, the system does not depend on the previous history of the system. For instance, the probability of developing a DE does not depend on whether the patient has previously experienced a DE or not. To allow the history to affect the behavior of the system, we would have to include additional state variables which would imply more complex higher-order systems. We are aware that to fully understand depression, it eventually will be necessary to incorporate such history-dependence. For instance, epidemiological studies have found evidence that adverse experience during childhood, such as sexual or physical abuse, neglect or loss of parents, is associated with substantial increase in the risk of developing depression [70]–[73]. Additionally, childhood trauma can change symptom patterns and the clinical course of MDD. For example, childhood trauma has been consistently associated with an early onset of depression [74], [75], as well as larger numbers of depressive episodes or more chronic depression [76], [77]. Moreover, childhood adverse experience has been associated with a decreased responsiveness to pharmacological treatment in patients with dysthymia and depression [78], [79]. However, not all forms of depression are associated with childhood adversity, and we may speculate that the high-risk sub-population in our model may partly include the group of depressive patients with a history of childhood trauma. Indeed, patients within the high-risk sub-population in our model tend to have more episodes, longer duration of episodes as well as more chronic episodes than those in the low-risk sub-population.

### Comparison between observations and model outputs

Our model offers an account for why AD treatment has only low rates of success and in some studies did not show superiority over placebo [36]–[38]. In our model, the distribution of DE durations in the treated patients is very broad with a peak at about 3 months and a long tail including episodes longer than 9 years. The properties of this distribution suggests two things. First, many studies were not able to see an effect of the AD treatment because the time window of observation was not long enough. Second, any effect of AD treatment is highly variable. Note that the parameters are identical for each simulation, so the widely different durations of depressive episodes did not emerge as a result of differences in the parameters. Furthermore, the risk of relapse seemed similar across heterogeneous groups of patients including those who had recently responded to treatment of an acute episode and those who had been successfully taking maintenance treatment for several months or even years [80]. Similarly, our modeling results indicate that AD treatment does not decrease the probability of developing another DE in the future.

### Practical implications

Our results imply that people who suffer from depression can be assigned to two sub-populations. The low-risk sub-population develops depression by chance, and those from this sub-population who suffer from MDD do not otherwise differ from those who never develop depression. The high-risk sub-population has an increased likelihood of developing depression, and tends to have more DEs during their life time, and longer DE durations. This prediction of our model may have relevance for clinicians, because it suggests that the patients who belong to the high-risk sub-population are at high risk of developing a chronic course of the disease. Moreover, this group of patients demands long-term treatment and regular check-ups after recovery.

We do not claim that our model is the final word on modeling the dynamics of depression. On the contrary, it has several apparent limitations some of which we have discussed above. The main goal of our article is to show the potential power of computationally modeling depression and the need for different quantitative data for understanding depression. We therefore hope that our modeling work will promote new empirical studies and/or reexaminations of existing data. In particular, we believe that it is important to monitor the disease progression in MDD on a day-to-day basis. The finite-state machine model that we developed here could be used to define the disease state of MDD more consistently and the operational criteria we suggested here might lead to improved design, interpretation, and comparison of studies of the natural course and clinical therapeutic trials. Ultimately, we hope that such efforts will lead to a clearer understanding of the nature of MDD.
